# Response to commentary by Skinner et al. on Regression to the Mean (RTM) in Burke et al.

**DOI:** 10.1186/s12966-015-0220-6

**Published:** 2015-05-07

**Authors:** Rachel M Burke, Christi Kay, Julie Gazmararian

**Affiliations:** Department of Epidemiology, Emory University, Atlanta, GA USA; HealthMPowers, Atlanta, GA USA

## Abstract

This letter is a response to commentary by Skinner et al. on an evaluation by Burke et al. of the HealthMPowers program, an elementary-school-based program developed to improve child health and wellness. In their commentary, Skinner et al. make the criticism that our results for changes in BMI-for-Age Z score were simply reflective of Regression to the Mean (RTM). In this response, we show that while some of our results are consistent with RTM, with adjustment we do still observe some small effects in BMI-for-Age Z score over the course of the school year. We conclude that while our evaluation was not definitive, we still believe that programs of similar design to HealthMPowers merit further rigorous study.

## Response to commentary

We thank Drs. Skinner et al. for their comments and feedback on our article [1]. Regression to the mean (RTM) is certainly an important statistical issue to consider, and one that should have been mentioned as a potential explanation to the results seen.

As mentioned in the original paper, this analysis had the severe limitation of lacking a control group. This is an effect of the fact that the HealthMPowers program was not designed as a research study, and the paper itself was conceived as an evaluation of the program based on existing data from its implementation. As Skinner et al. explain, one-group designs are particularly vulnerable to the potential effects of RTM. Nonetheless, it would be incorrect to state that we found “no significant reduction in BMI [Body Mass Index] z-scores in the total sample”; in fact, analysis of the total sample indicated a highly significant (p < 0.0001), though modest decrease of 0.06 in BMI-for-Age Z score over the course of the school year. Significance was maintained (p < 0.0001), though the effect further attenuated to a 0.04 decrease, when constrained to children normal-weight at baseline. Generally, the high correlation coefficient (rho = 0.96) between pre and post BMI-for-Age Z score would suggest that the effect of RTM, and indeed any observed one-year effect of the program, should be modest. In response to this feedback, we also performed an analysis of the potential effect of RTM on the published results (a subset of obese children stratified on grade and sex), based on formulae in Davis [2]. This analysis indicated that, while some RTM can be expected and could explain the results, there may still be a true, though again quite modest, effect observable in some subgroups (observed differences in BMI-for-Age Z score ranging 0.05 – 0.12, compared to 0.08 expected difference).

Even though the evidence may not conclusively support an effect of the HealthMPowers program on body composition as measured via change in BMI-for-Age Z score, we believe that programs of this design still have merit and warrant further rigorous evaluation. Our analysis was suggestive of improvements in student knowledge and behaviors, PACER performance, and self-assessed improvements in the school environment, all of which are important to holistically improving child health and wellness. We hope that our publication serves to inspire the development and evaluation of other similarly designed school-based programs that focus not only on student education and physical activity, but also on teacher and parent involvement. It is clear that more research is needed in this area, with special focus on randomized trials where possible, given the difficulties involved in obesity-prevention research. Our research team is currently in the initial stage of implementing a randomized controlled trial of the HealthMPowers program and its effect on the relationship between physical activity, physical fitness, and academic achievement. This planned research will help to more definitively identify whether and to what magnitude a comprehensive school-based program can effect change in student health and academic outcomes.

## References

[1] Burke RM, Meyer A, Kay C, Allensworth D, Gazmararian JA (2014). A holistic school-based intervention for improving health-related knowledge, body composition, and fitness in elementary school students: an evaluation of the HealthMPowers program. Int J Behav Nutr Phys Act.

[2] Davis CE (1976). The effect of regression to the mean in epidemiologic and clinical studies. Am J Epidemiol.

